# Letter to the Editor for “Metronomic Chemotherapy in Pediatric Neuroblastoma”

**DOI:** 10.1002/pdi3.70003

**Published:** 2025-04-17

**Authors:** Ana Ivanovski, Dimitrije Nikolic, Ana Cirovic, Aleksandar Cirovic

**Affiliations:** ^1^ Faculty of Medicine University of Belgrade Belgrade Serbia; ^2^ Neurology Department University Children's Hospital Belgrade Serbia; ^3^ Faculty of Medicine Institute of Anatomy University of Belgrade Belgrade Serbia

Dear Editor,

Jiang et al. recently discussed the promising effects of metronomic chemotherapy for the treatment of pediatric neuroblastoma in their paper, which we read with great interest [1]. We agree that Kerbel and Hanahan have made substantial contributions to the implementation and development of metronomic chemotherapy [2, 3]. However, as far as we are aware, Ivanovski et al. in 1991 reported a case in which a child with fibrolamellar hepatocarcinoma and solitary bilateral pulmonary metastases was treated over a prolonged period with low‐dosage oral chemotherapeutic agents [4]. Therefore, we once again congratulate the authors on this interesting paper and would like to highlight that Ivanovski et al. may be considered pioneers of metronomic chemotherapy.

## Ethics Statement

The authors have nothing to report.

## Consent

The authors have nothing to report.

## Conflicts of Interest

The authors declare no conflicts of interest.

## Permission to Reproduce Material From Other Sources

The authors have nothing to report.

## Data Availability

Data sharing is not applicable to this article as no new data were created or analyzed in this study.
